# The impact of anxiety on Chinese as a second language achievement: a meta-analytic perspective

**DOI:** 10.3389/fpsyg.2025.1620275

**Published:** 2025-09-15

**Authors:** Peng Qiao, Zhicheng Yuan, Shuhong Li

**Affiliations:** ^1^Department of Humanities, Hunan City University, Yiyang, Hunan, China; ^2^Department of Foreign Languages, Ocean University of China, Qingdao, Shandong, China

**Keywords:** anxiety, second language acquisition, Chinese achievement, control-value theory, meta-analysis

## Abstract

In light of the growing global enthusiasm for learning Chinese, understanding the psychological factors influencing Chinese language learners—particularly the impact of anxiety on second language Chinese achievement (hereafter “Chinese achievement”)—is essential for improving teaching effectiveness. However, the specific effects and magnitude of this relationship remain a topic of academic debate. Grounded in the Control-Value Theory, this study employs a meta-analytic approach to synthesize findings from 23 empirical studies, encompassing 58 independent effect sizes and a total of 4,191 participants. The results reveal a small-to-moderate negative correlation between anxiety and Chinese achievement. Moderator analyses indicate that language proficiency level and the proportion of female learners significantly influence this relationship. Specifically, the negative impact of anxiety is most pronounced among beginners, followed by advanced, intermediate, and mixed-proficiency learners. Additionally, anxiety has a stronger detrimental effect on Chinese achievement in learner groups with a higher proportion of females. However, publication language, participant region, learning context, language skills, and achievement measurement did not exhibit significant moderating effects. These findings provide valuable implications for international Chinese language education, highlighting the importance of a student-centered teaching approach, optimized instructional strategies, and enhanced teaching quality.

## 1 Introduction

As an increasing number of people worldwide are learning Chinese, the field of Chinese as a Second Language (CSL) education is encountering unprecedented opportunities and challenges. Among these, an in-depth exploration of learners’ psychological factors has become an urgent and indispensable necessity. Currently, the global spread of Chinese language education has reached an unprecedented level, with over 25 million individuals actively learning Chinese and nearly 200 million having studied or used the language (People’s Daily Overseas Edition, 2022). This large and diverse learner population poses challenges for optimizing instructional strategies and allocating resources in CSL education.

Given the growing learner base, accurately identifying learners’ psychological characteristics—particularly the deep-seated psychological factors that influence learning outcomes—has become essential for enhancing instructional quality and ensuring the efficient utilization of educational resources. Since the late 1990s, influenced by the humanistic paradigm, scholars have increasingly focused on language anxiety among Chinese language learners. In recent years, within the framework of the “emotional turn,” the complex relationship between anxiety and CSL achievement has been explored from new perspectives, gaining renewed significance (Chen, 2018; Li and Dewaele, 2021). A deeper understanding of this relationship holds immense value for implementing student-centered educational approaches, refining teaching strategies, improving instructional effectiveness, and informing the development of educational strategies.

However, the existing literature on the direction and degree of the impact of anxiety on Chinese achievement is not consistent. Although some theoretical reviews (e.g., Yao et al., 2022; Xu, 2018) provide valuable insights into the phenomenon of language anxiety, they mainly focus on theoretical discussions rather than empirical synthesis. To address this gap, this study systematically integrated existing empirical research on the relationship between anxiety and Chinese achievement using meta-analysis. Meta-analysis, with its unique quantitative synthesis ability, enables researchers to go beyond the limitations of individual studies and provide a more comprehensive, systematic, and scientific understanding of this phenomenon. As Oswald and Plonsky (2010) stated, meta-analysis “not only reviews the historical trajectory of applied linguistics research but also guides its future development.” This study aims to provide empirical evidence to support the second language Chinese teaching method and further improve teaching effectiveness.

## 2 Literature review

### 2.1 Anxiety and Chinese language learning

As a common psychological phenomenon in Chinese classroom teaching, anxiety is a highly arousing, outcome-oriented negative emotion. Such an emotion is closely related to learners’ expectations of success or failure (Yao et al., 2022; Pekrun et al., 2007). Anxiety manifests itself in the form of activation of the autonomic nervous system, which leads to increased tension and worry (Spielberger, 1983). In the field of second language acquisition (SLA), foreign language anxiety (FLA) has been defined as a complex, multidimensional phenomenon. FLA manifests itself in linguistic contexts in the form of fear, interference with cognitive processing, and effects on performance (Horwitz, 2016; Rasool et al., 2023a). FLA includes the interrelated dimensions of self-perception, language beliefs, emotional reactions, and classroom behavior. Recent meta-analyses (e.g., Chen, 2025) have emphasized the differential effects of anxiety on specific language skills, highlighting the need for investigations into the underlying mechanisms, and teaching effectiveness.

Although early research had a relatively limited understanding of anxiety, contemporary studies distinguish between trait anxiety (a stable tendency), state anxiety (a situational condition; Baran-Łucarz, 2022), skill-specific anxiety (e.g., speaking: Demirdöken and Okur, 2022; listening: Chang, 2008; writing: Rasool et al., 2023a; An and Li, 2024), and task-related anxiety (Wang et al., 2024). This study synthesized the overall effect of anxiety but acknowledged the limitations related to specificity of measurement.

In contemporary SLA research, the Control-Value Theory (Pekrun et al., 2007) has become the core framework for understanding achievement emotions. This theory posits that achievement emotions are determined by two key appraisals: control appraisal (the perceived ability to successfully complete a task) and value appraisal (the subjective importance assigned to a task). When learners experience moderate levels of control but hold negative evaluations of the task’s value—particularly excessive concern over failure—anxiety is likely to arise. Anxiety, in turn, impacts cognitive processing (e.g., attention allocation, information retention, memory consolidation), learning motivation, and ultimately academic achievement.

With the “affective turn” in the field of SLA, there has been an increased focus on the role anxiety plays in language achievement (Li and Dewaele, 2021). However, research findings remain controversial (Yao et al., 2022). Some studies report a positive correlation between anxiety and language achievement (e.g., Liang and Quan, 2016; Zhang and Yang, 2011; Yang, 2010), suggesting that moderate anxiety can be a motivator. In contrast, other studies have shown negative correlations (e.g., Wang and Du, 2020; Liu, 2018; Luo, 2015), indicating that anxiety diminishes motivation and confidence, which negatively affects performance. In addition, the reported effect sizes vary considerably, ranging from large (e.g., Zhou, 2023), to medium (e.g., Zhang and Wang, 2002; Zhang and Yang, 2011), and small (e.g., Liu, 2018; Luo, 2015).

Given the complex relationship between anxiety and Chinese achievement, this study used meta-analysis to conduct a comprehensive and systematic examination. As a statistical technique for re-analyzing the results of quantitative studies, meta-analysis aims to aggregate statistical indicators from multiple studies to accurately assess the true correlation between variables. The method has demonstrated great applicability and value in a variety of disciplines, including medicine, psychology, education, and linguistics. Meta-analysis has significant advantages in terms of objectivity compared to traditional literature review methods. Not only can it rigorously address sampling errors and explain statistically insignificant results, but it can also effectively reduce analytical biases that may be caused by individual studies or subjective interpretations. Notably, meta-analysis helps identify systematic patterns in variable relationships, thus providing more nuanced insights into complex phenomena (Plonsky and Oswald, 2015; Vuogan and Li, 2023). Although existing meta-analysis studies in the field of SLA have mainly focused on the English language domain, meta-analysis studies in the context of CSL remain relatively scarce (Yao et al., 2022). Therefore, the present study aims to explore the relationship between anxiety and Chinese achievement from a scientifically rigorous and comprehensively integrated methodological perspective, with a view to providing empirical support for the practice of CSL.

### 2.2 Potential moderator variables

The inconsistency in the existing research results on the relationship between anxiety and Chinese achievement mentioned above may stem from diversities in research characteristics, such as research background, participant demographics, and research design (Vuogan and Li, 2023). Based on a literature review, seven key moderating variables were identified as potential factors underlying the complex relationship between anxiety and Chinese achievement.

A key factor in meta-analysis is the potential bias introduced by publication language. Compared to non-English journals, English journals may be more inclined to publish research with more favorable or statistically significant results, leading to so-called “English bias” or “Tower of Babel bias” (Egger et al., 1997; Grégoire et al., 1995). To minimize this bias, this study not only included English and Chinese publications, but also included the language of publication as a moderating variable to test its potential impact. Second, the region where the participants were located is also worth considering. Given that Chinese is characterized by a pinyin and ideographic writing system, proximity between language and culture may influence language transfer. For example, learners from East and Southeast Asia have native languages that share common historical and cultural roots with Chinese. As a result, this may motivate learners to experience positive language transfer, thereby reducing their anxiety levels. In contrast, learners from Indo-European backgrounds (e.g., Western learners) may face greater linguistic structural differences, which may impede language transfer and thus increase anxiety (Yao et al., 2022). Based on this premise, it is hypothesized that Asian learners may exhibit lower levels of anxiety, thereby reducing the impact of anxiety on Chinese achievement.

Third, the learning context may be a potential moderating factor. In China, international students have an immersive language environment, sufficient learning resources, and opportunities for direct interaction with native speakers, which may help reduce the impact of anxiety on language learning. In contrast, students learning Chinese in their home countries may face greater challenges due to limited resources and fewer opportunities for practice, which may lead to higher anxiety levels and reduce learning efficiency. Fourth, language level may be associated with anxiety. Intermediate and advanced learners usually have longer exposure to the target language, which may help reduce anxiety levels (Basith et al., 2019). Therefore, it can be assumed that intermediate and advanced learners may have lower anxiety levels than beginners, which may have less impact on their language achievement.

Fifth, since different language skills may impose different cognitive demands on learners, they may act as moderating variables. Specifically, listening and speaking skills require real-time processing and have higher cognitive load requirements. Therefore, this is more likely to lead to higher anxiety levels, which will have a greater impact on language performance. In contrast, reading and writing skills allow for more forethought and impose lower cognitive demands, potentially triggering lower levels of anxiety, leading to potentially more limited effects on achievement outcomes (Teimouri et al., 2019). Sixth, the measurement of language achievement may produce different results. This study uses HSK scores, course scores, and self-assessment scores as moderating variables to assess the consistency of the impact of anxiety on Chinese achievement under different assessment criteria, thereby ensuring the robustness and generalizability of the research results. Seventh, female ratio may moderate the relationship between anxiety and Chinese achievement. Studies have shown that female learners tend to show higher anxiety levels in SLA environments, which may be because they are more afraid of making mistakes and have lower confidence in the use of the target language (Park and French, 2013; Dewaele et al., 2016). This tendency is further exacerbated by gender socialization processes, which amplify the emotional impact of errors and cause female learners to have stronger anxiety reactions (Rasool et al., 2023b). Previous meta-analysis results also support this pattern, showing that female learners have significantly higher levels of foreign language anxiety than male learners (Piniel and Zólyomi, 2022). Therefore, in learning environments with a higher proportion of female learners, the impact of anxiety on language achievement may be more significant.

In summary, given the importance of these variables as potential moderators of the relationship between anxiety and Chinese achievement, this meta-analysis will examine the potential effects of factors, including publication language, participant region, learning context, proficiency level, language skill, achievement measurement, and female proportion. By synthesizing existing studies, this research aims to clarify the complex mechanisms through which anxiety affects Chinese achievement.

## 3 Research methodology

### 3.1 Research questions

In this study, the empirical research on the relationship between anxiety and Chinese achievement will be quantitatively and systematically analyzed through meta-analytic methods. It aims to reveal the intrinsic connection between these two variables. Specifically, this study attempts to address the following research questions:

Question 1: What is the overall effect size of the relationship between anxiety and Chinese achievement?

Question 2: What moderating variables significantly affect the relationship between anxiety and Chinese achievement?

### 3.2 Literature search

To ensure the comprehensiveness of the sample, both published literature and “grey literature” were included in this study to minimize possible publication bias (Norris and Ortega, 2006). Following the meta-analysis guidelines proposed by Plonsky and Brown (2015), the literature search was conducted in three stages. First, a systematic search was conducted in Chinese and international academic databases, including the Education Resources Information Center (ERIC), Web of Science (WOS), Linguistics and Language Behavior Abstracts (LLBA), ProQuest, Google Scholar, and China National Knowledge Infrastructure (CNKI). The search employed keywords such as “anxiety” and “Chinese achievement” and utilized the search string “(Anxiety OR anxi*) AND (achievement OR performance OR proficiency) AND (language OR SL OR FL OR Chinese).” Second, reference lists of relevant review articles and studies were manually reviewed to supplement and refine the search results. Finally, drawing on Perera and DiGiacomo’s (2013) approach, a targeted search of major journals in the relevant field was conducted to further reduce the risk of missing relevant literature. Based on the comprehensive search strategy, 961 relevant studies were initially identified.

### 3.3 Inclusion criteria

**Figure 1:**
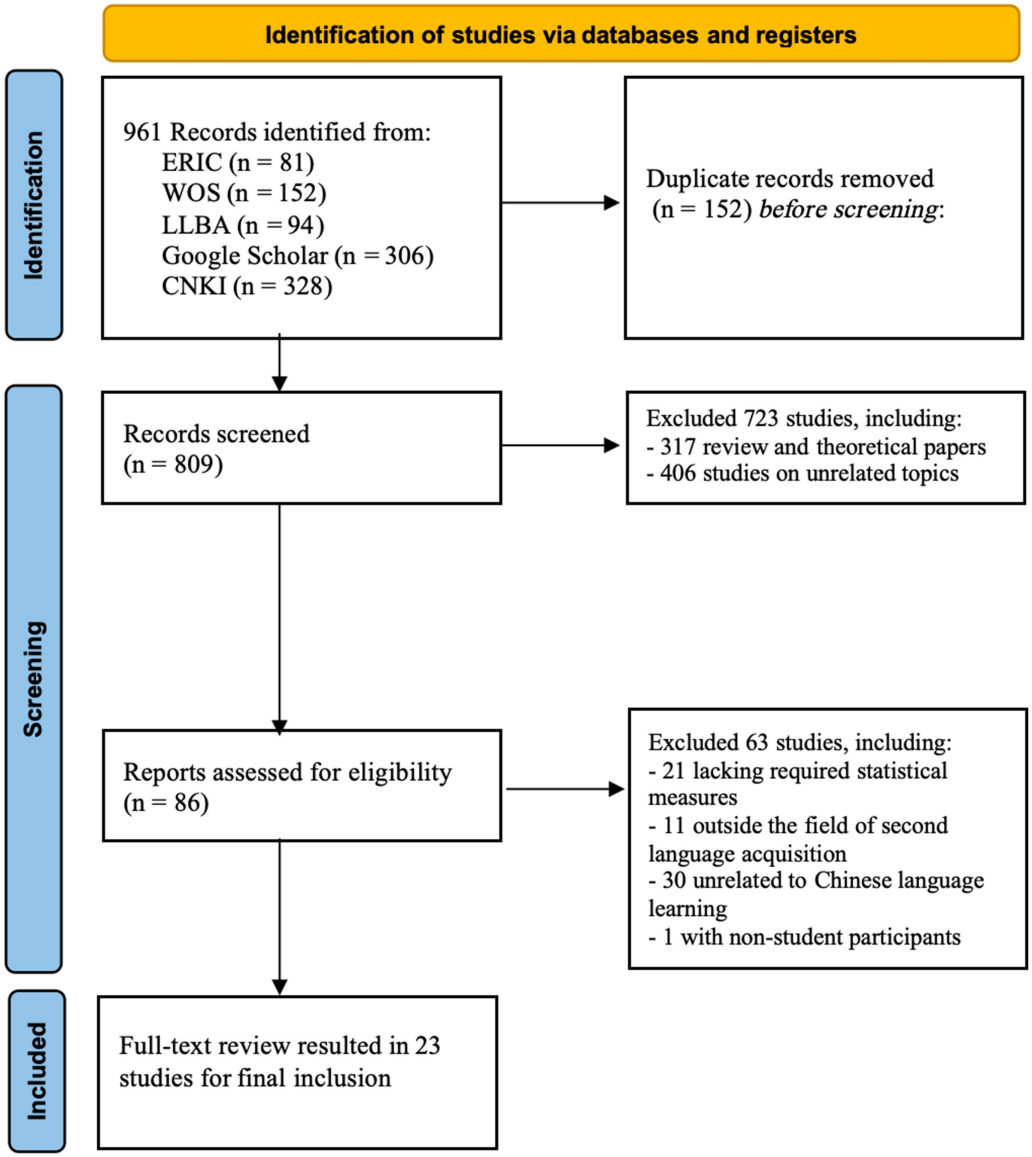
Flowchart of the literature selection process for meta-analysis.

Four inclusion criteria were identified based on the study domain, measurement instrument, data format, and participant characteristics: (1) the study must be relevant to the CSL domain; (2) the study must report a measurement instrument for both anxiety and Chinese achievement; (3) it must report correlation coefficients (*r*) or provide statistical data (e.g., *t*-values or *F*-values) that can be converted to effect sizes for the correlation between anxiety and Chinese achievement; (4) the participants must be in the student population; and (5) the study must be published in a peer-reviewed journal to ensure research quality. After applying these inclusion criteria and screening, 23 studies were retained and included in the meta-analysis. The detailed screening process is shown in Figure 1.

### 3.4 Overview of included studies

Among the 23 included studies, 19 were published in Chinese-language journals, while 4 appeared in English-language journals. In terms of temporal distribution, 9 studies were published before 2016, whereas 14 were published in 2016 or later. The total sample size across all studies was 4,191 participants, covering multiple regions, including Asia, Europe, and Africa. The breadth of geographical and temporal distribution, as well as the scale of the participant pool, provides a robust empirical basis for examining the relationship between language anxiety and Chinese achievement among learners.

Additionally, the included studies exhibit the following characteristics: (1) The participants’ proficiency levels are broadly representative, encompassing beginner, intermediate, and advanced learners, thereby ensuring the comprehensiveness and reliability of the findings; (2) existing research tends to focus on international students at the university level, while studies on primary and secondary school learners studying Chinese in their home countries remain relatively scarce; (3) the assessment of Chinese achievement in the included studies is multidimensional, covering not only overall Chinese achievement but also listening, speaking, reading, and writing skills, which contributes to a more comprehensive understanding of the impact of anxiety on various aspects of language achievement.

### 3.5 Literature coding

Following multiple rounds of refinement, the study coded the literature based on nine key aspects: (1) study details (title, author, year of publication, language of publication, publication type); (2) sample size; (3) effect size (*r*); (4) proportion of female participants; (5) language proficiency level (beginner, intermediate, advanced, mixed, not reported); (6) participants’ geographic background (Asia countries, western countries, mixed); (7) learning context (international students in China, domestic learners); (8) language skills (mixed, listening, speaking, reading, writing); (9) achievement measurement (HSK test, course grades, self-reported scores). The coding scheme is provided in Appendix B. If a study reported effect size data for different subgroups (e.g., beginner, intermediate, and advanced learners), the data were independently coded following Lipsey and Wilson’s (2001) recommendations, resulting in 58 independent effect sizes.

The coding process was independently conducted by two trained researchers, both of whom are experts in the fields of Second Language Acquisition and Applied Linguistics. All studies were double-coded to ensure consistency across the entire dataset. The inter-rater reliability was high (Cohen’s Kappa = 0.95). Any discrepancies were resolved through discussion and re-examination of the original studies. When consensus could not be reached, a third expert served as an adjudicator. This process ensured the accuracy and reliability of the coding and was carried out with methodological rigor.

### 3.6 Data analysis

The study began with data coding using Statistical Products and Services Solution (SPSS) v27.0, followed by meta-analysis using Comprehensive Meta-Analysis Software (CMA) v3.0. Given the heterogeneity of the included studies, this study used a random effects model rather than a fixed effects model to ensure the robustness of the analyses (Vuogan and Li, 2023). Regarding the interpretation of effect sizes, the present study followed the criteria proposed by Plonsky and Oswald (2014) that are more appropriate for SLA research, categorizing effect sizes as small (|0.25|), medium (|0.40|) and large (|0.60|). In order to justify model selection and determine the necessity of moderator variable analyses, a series of heterogeneity tests, including *I^2^*, τ^2^, and *Q*-tests, were conducted to assess variance dispersion and between-study differences (Lipsey and Wilson, 2001).

Given the potential impact of publication bias on the results of this meta-analysis, this study used several methods to mitigate and assess bias. First, no restriction was placed on publication date during the literature search, and “grey literature” was included wherever possible. Second, publication bias was assessed using the funnel plot, classic fail-safe *N* and Kendall’s tau statistics. The fail-safe *N* indicates the number of unpublished studies required to overturn the results of the meta-analysis. The threshold was set at 5 *k* + 10 (where *k* indicates the number of effect sizes). If *N* significantly exceeds this benchmark, the results of the meta-analysis can be considered robust and unlikely to be overturned by unpublished studies; the higher the *N*, the lower the likelihood of bias affecting the results (Rothstein, 2008). In addition, a non-significant result from a Kendall tau analysis would (*p* > 0.05) indicate the absence of substantial publication bias. Besides, a sensitivity analysis (leave-one-out procedure) was conducted to evaluate the robustness of the results by systematically omitting each included study one at a time (Patsopoulos et al., 2008). Finally, subgroup analyses and meta-regression analyses were performed to further explore potential moderating variables.

## 4 Results

### 4.1 Heterogeneity test

The goodness-of-fit statistics for the heterogeneity test are presented in Table 1. The results indicate a significant goodness-of-fit statistic (*Q* = 1609.10, *p* < 0.001), with τ^2^ = 0.39. The *I^2^* value indicates that 96.46% of the variance in effect sizes is attributable to factors beyond mere sampling error, such as differences in study design, sample characteristics, and contextual variables (Vuogan and Li, 2023). This degree of heterogeneity is considered substantial (*I^2^* ≥ 75%) and suggests considerable variability across studies (Vuogan and Li, 2023). Accordingly, moderation analyses were conducted under a random effects model to further explore the potential sources of this variation (Li and Wang, 2018).

**Table 1:** Heterogeneity test results for anxiety and Chinese achievement correlation.

Effect	Heterogeneity test					
	*k*	*Q*	*df*	Sig.	τ^2^	*I* ^2^
Anxiety-Chinese achievement	58	1609.10	57	0.00	0.39	96.46

### 4.2 Publication bias test

**Figure 2:**
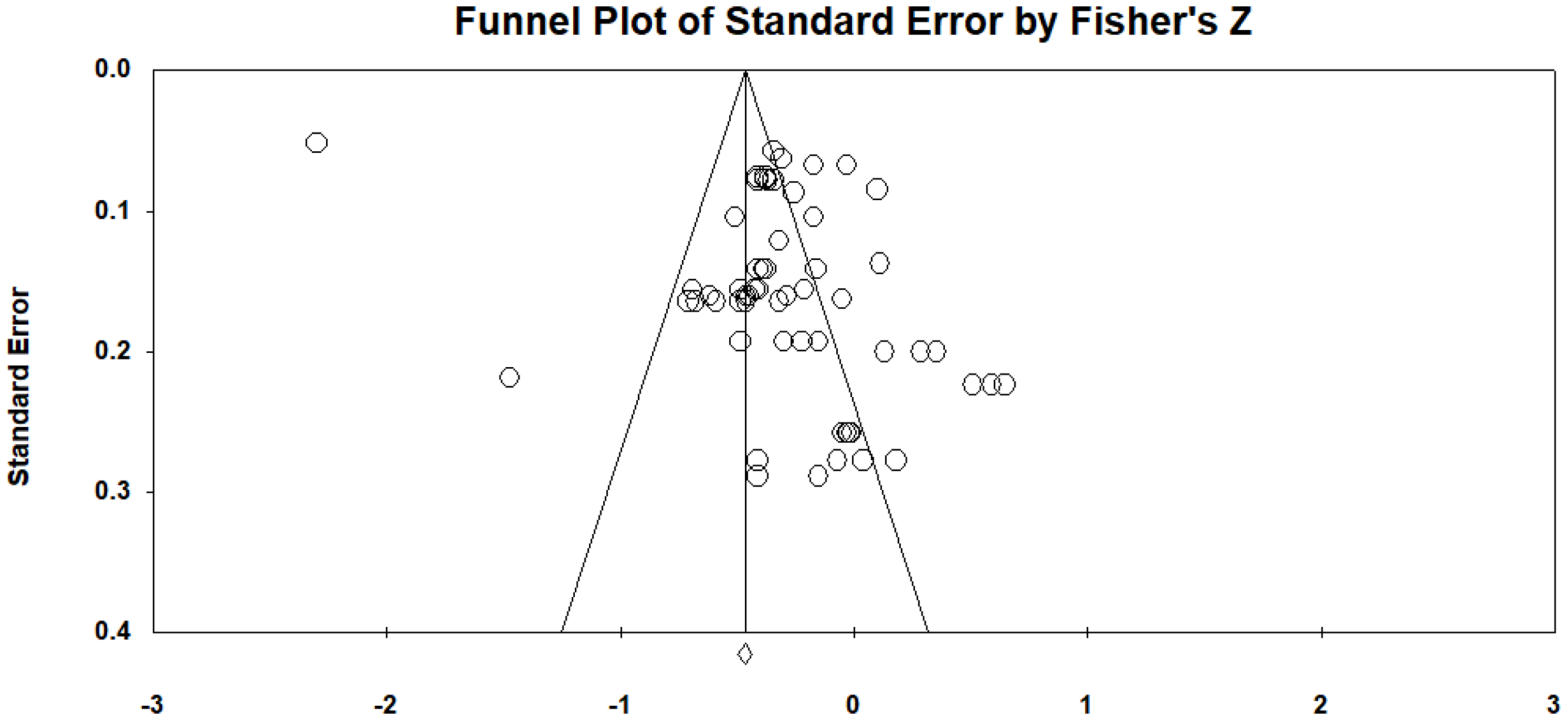
Funnel plot.

To assess the potential presence of publication bias—which refers to the systematic underrepresentation of non-significant or unfavorable results (Little and Rubin, 2019)—we employed both visual and statistical approaches. First, a funnel plot was constructed to visualize the relationship between effect sizes and their standard errors (see Figure 2). While the plot shows that most studies cluster around the mean effect size, a degree of asymmetry is apparent, particularly with fewer studies exhibiting small negative effects at higher standard errors. This visual pattern may reflect potential publication bias or small-study effects. However, given the known limitations and subjectivity of visual inspection alone (Terrin et al., 2003), we conducted several complementary quantitative analyses to substantiate the interpretation. Second, the Classic Fail-safe *N* analysis yielded a value of 6,435, indicating that over six thousand unpublished studies with null results would be required to bring the overall effect size to non-significance. This substantially exceeds the recommended threshold of 5 *k* + 10 (Rothstein, 2008), suggesting strong robustness against potential unpublished null findings. Third, Kendall’s rank correlation test between standardized effect sizes and their standard errors yielded a non-significant result (*p* > 0.05), indicating no statistically significant evidence of publication bias. Fourth, we conducted a leave-one-out sensitivity analysis, which revealed that the pooled effect size remained stable and statistically significant across all iterations, indicating that the results are not unduly influenced by any single study.

Taken together, although minor asymmetry was observed in the funnel plot, the convergence of multiple robust statistical diagnostics suggests that publication bias is unlikely to meaningfully compromise the validity or stability of the meta-analytic findings.

### 4.3 Overall effect size between anxiety and Chinese achievement

To address Research Question 1, random effects models were used to estimate overall weighted effect sizes. The results showed a small to moderately significant negative correlation between anxiety and Chinese achievement (*r* = −0.28, *p* < 0.001; Plonsky and Oswald, 2014), with 95% confidence intervals (CIs) ranging from −0.42 to −0.12. This result suggests that higher levels of anxiety are associated with lower Chinese achievement. A forest plot illustrating the distribution of effect sizes across studies is provided in Appendix A.

### 4.4 Moderating variables in the anxiety–Chinese achievement relationship

To address Research Question 2, moderator analysis was conducted using subgroup analysis for categorical variables and meta-regression analysis for continuous variables. Based on the data in Table 2, the following conclusions were drawn:

**Table 2:** Moderator analysis of the relationship between anxiety and Chinese achievement.

Categorical variables	95% CI				
	*k*	*r*	Lower	Upper	*Q _between_*
Publication language					0.00
Chinese	51	−0.28	−0.46	−0.08	
English	7	−0.28	−0.37	−0.18	
Region of participants					2.15
Western countries	2	−0.29	−0.40	−0.18	
Asian countries	39	−0.22	−0.32	−0.11	
Mixed	17	−0.43	−0.68	−0.10	
Learning context					0.18
International students in China	56	−0.28	−0.43	−0.10	
Domestic learners	2	−0.32	−0.39	−0.24	
Proficiency level					19.46^***^
Beginner	10	−0.39	−0.48	−0.29	
Intermediate	3	−0.14	−0.37	0.11	
Advanced	5	−0.33	−0.46	−0.18	
Mixed	25	−0.09	−0.20	0.02	
Language skill					0.84
Speaking	11	−0.42	−0.77	0.14	
Listening	12	−0.28	−0.40	−0.14	
Writing	7	−0.28	−0.38	−0.17	
Reading	12	−0.27	−0.51	−0.00	
Mixed	16	−0.23	−0.34	−0.14	
Achievement measurement					1.15
HSK test	36	−0.30	−0.53	−0.02	
Course grades	17	−0.29	−0.36	−0.22	
Self-reported scores	5	−0.34	−0.40	−0.28	

Continuous variable	95% CI				
	Point estimate	SE	Lower	Upper	*Q*_between_
Female proportion	0.62	0.17	0.28	0.97	12.87^***^

Due to the absence of reported participant proficiency levels in some studies, 15 independent effect sizes with unspecified learner proficiency levels have been excluded from the “Proficiency Level” subgroup analysis.

First, publication language was not a significant moderator of the anxiety - Chinese achievement relationship (*Q* = 0.00, *p* > 0.05). The effect sizes reported in Chinese-language journals (*r* = −0.28) and English-language journals (*r* = −0.28) were consistent, indicating that the relationship remains stable across Chinese and international publications. Second, participant region did not significantly moderate the relationship (*Q* = 2.15, *p* > 0.05). However, the effect size was slightly higher for mixed-population samples (*r* = −0.43) compared to Western (*r* = −0.29) and Asian (*r* = −0.22) samples, suggesting that anxiety had the strongest impact on Chinese achievement among mixed-population learners, followed by Western and Asian learners.

Third, learning context was also not a significant moderator (*Q* = 0.18, *p* > 0.05), though the correlation in the international student group (*r* = −0.28) was slightly lower than that of domestic learners (*r* = −0.32), indicating that anxiety had a slightly weaker effect on Chinese achievement for international students. Notably, language proficiency level significantly moderated the anxiety–Chinese achievement relationship (*Q* = 19.46, *p* < 0.001). Anxiety had the strongest effect among beginner learners (*r* = −0.39), followed by advanced (*r* = −0.33), intermediate (*r* = −0.14), and mixed-level learners (*r* = −0.09). Additionally, the impact of anxiety across different language skills was not significant (*Q* = 0.84, *p* > 0.05). However, the effect was most pronounced in speaking assessments (*r* = −0.42), followed by listening (*r* = −0.28), writing (*r* = −0.28), reading (*r* = −0.27), and mixed skills (*r* = −0.23).

Similarly, achievement measurement was not a significant moderator (*Q* = 1.15, *p* > 0.05), but the correlation was strongest for self-reported grades (*r* = −0.34), followed by HSK test (*r* = −0.30) and course grades (*r* = −0.29). Of particular importance, the female proportion significantly moderated the anxiety–Chinese achievement relationship (*Q* = 12.87, *p* < 0.001), indicating that the relationship was stronger in groups with a higher percentage of female participants. In summary, proficiency level and female proportion significantly moderated the relationship between anxiety and Chinese achievement, whereas publication language, participant region, learning context, language skills, and achievement measurement did not exhibit significant moderating effects.

## 5 Discussion

### 5.1 Relationship between anxiety and Chinese achievement

A meta-analysis of 23 studies encompassing 4,191 participants revealed a significant negative correlation of small-to-moderate magnitude between anxiety and Chinese achievement (*r* = −0.28). This finding supports the Control-Value Theory (Pekrun et al., 2007), confirming that emotions influence academic achievement. Specifically, anxiety, as a high-arousal, outcome-focused negative emotion, impairs Chinese language learning, thereby undermining language acquisition.

A comparison with meta-analytic findings in English learning (Teimouri et al., 2019; *r* = −0.33) suggests that the impact of anxiety on language achievement varies across target languages: anxiety exerts a stronger negative influence on academic achievement in English learning than in Chinese learning. This discrepancy may be attributed to the external motivational pressures associated with English as a global lingua franca, where learners frequently experience heightened anxiety due to academic and career expectations. The societal emphasis on English proficiency exacerbates learners’ psychological burden, intensifying anxiety and further impairing English achievement. In contrast, learners of Chinese may be more intrinsically motivated, particularly through cultural interest, which mitigates the negative impact of anxiety on Chinese achievement (Wang and Du, 2020).

### 5.2 Moderating variables in the relationship between anxiety and Chinese achievement

Moderator analyses indicated that proficiency level and female proportion significantly moderated the relationship between anxiety and Chinese achievement, while publication language, participant region, learning context, language skills, and achievement measurement did not exhibit significant moderating effects, despite notable subgroup differences. These findings are discussed as follows.

Proficiency level emerged as a significant moderator, profoundly influencing the anxiety-achievement relationship. The effect was most pronounced among beginner learners, followed by advanced, intermediate, and mixed-level learners. Beginners’ vulnerability aligns with Cognitive Load Theory (Sweller, 2020), while advanced learners’ anxiety resurgence reflects complex pragmatic demands (Dewaele et al., 2018). This U-shaped pattern contrasts linear “anxiety reduction” hypotheses (Basith et al., 2019). A plausible explanation is that beginners, due to their limited linguistic foundation, are more susceptible to anxiety triggered by course difficulty, while advanced learners encounter increased cognitive challenges in language application. Intermediate learners, benefiting from both a solid foundation and moderate learning difficulty, experience relatively lower anxiety, resulting in a weaker negative impact on achievement. Additionally, female proportion significantly moderated the relationship, indicating a stronger negative impact of anxiety on Chinese achievement in groups with a higher female proportion. This aligns with previous research (Park and French, 2013; Dewaele et al., 2016). According to Gender Role Socialization Theory, gender roles become internalized through socialization. Thus, female learners, influenced by societal expectations, tend to exhibit heightened perfectionism. Consequently, they are more prone to anxiety when faced with unattainable goals, which adversely affects their language learning outcomes (Ghorbandordinejad, 2014).

Publication language was not a significant moderator, reinforcing the consistency of this relationship across studies published in Chinese and English. This finding counters concerns regarding potential “English bias” and “Tower of Babel bias” (Egger et al., 1997; Grégoire et al., 1995), supporting the objectivity and scientific rigor of the present study. Given the limited empirical meta-analytic examinations of these biases, future research should further investigate their presence to enhance the reliability of educational policy formulation.

Although the participant region did not emerge as a significant moderator, subgroup analyses revealed nuanced regional differences: the anxiety-achievement relationship was weakest among Asian learners, followed by Western learners, and strongest in mixed-region samples. This corroborates findings by Yao et al. (2022) and Fan (2009), who suggested that linguistic and cultural proximity among Asian learners facilitates positive transfer effects, thereby mitigating the detrimental impact of anxiety. The weaker links between anxiety and achievement in Asian learners might also indicate conformity to cultural schemas—for instance, shared Confucian values that promote literacy transfer (Stankov, 2010). Conversely, Western learners, facing a starkly different linguistic and cultural background, experience greater negative transfer and heightened anxiety, which significantly impairs their Chinese achievement. The strongest effect size observed in mixed-region samples may stem from the broad diversity of study participants, particularly those from Africa and Oceania. Learners from these regions, being less familiar with the Chinese linguistic and cultural system, tend to experience higher anxiety levels, contributing to the overall effect size. However, the limited representation of African learners in primary studies may obscure the true cultural gap effects, underestimating the role of anxiety in these populations. Given the paucity of research on African and Oceanian learners, future studies should explore these populations in greater depth to provide more precise and comprehensive theoretical and practical insights for global Chinese language education, ensuring equitable and high-quality learning opportunities worldwide.

The results showed that learning context was not a significant moderator. However, the negative impact of anxiety on achievement was weaker for international students in China compared to domestic learners. This difference may be attributed to the fact that the immersive learning environment experienced by international students alleviated anxiety, thus mitigating the negative impact of anxiety on Chinese language learning. In addition, although language skills did not emerge as a significant moderating variable, it can be known that anxiety had the greatest impact on speaking achievement. This finding is in line with Teimouri et al. (2019), which emphasized that speaking tasks are cognitively demanding, time-critical and prone to anxiety. Furthermore, the achievement measurement did not significantly affect the relationship of the variables, suggesting that the observed effects were stable across assessment methods. However, a noteworthy finding was that anxiety had the strongest negative correlation with self-reported achievement, suggesting that anxious learners tend to underestimate their language proficiency. This is consistent with previous research (Teimouri et al., 2019) and emphasizes the subjective impact of anxiety on learners’ self-ratings.

In conclusion, this systematic meta-analysis provides strong evidence that anxiety has a significant effect on Chinese achievement. Such results challenge the field’s traditional emphasis on cognitive determinants. Furthermore, the broad consistency of findings across different research backgrounds (e.g., language skills, type of assessment, learner region, and publication language) underscores the cross-situational relevance of this relationship and emphasizes the need to adopt pedagogical approaches that focus on anxiety in international Chinese language education.

## 6 Conclusion and implications

This study synthesized findings from 23 studies involving 4,191 participants through a meta-analytic approach, revealing a small-to-moderate negative correlation between anxiety and Chinese achievement. Proficiency level and female proportion significantly moderated this relationship, while publication language, region of participant, learning context, language skills, and achievement measurement did not exert significant moderating effects.

These findings yield several implications for CSL instruction. First, they underscore the importance of addressing anxiety as a non-cognitive factor in language learning. In particular, more targeted support should be provided to underrepresented learner groups—such as those from Africa and Oceania—to enhance both their academic outcomes and emotional well-being. Second, given the demonstrated adverse effects of anxiety on CSL achievement, it is essential that educators develop the capacity to identify and respond to learners’ emotional states. Intervention strategies which include structured emotion regulation training, competitive task design, music-assisted relaxation, and enhanced teacher-student rapport may help to create a more emotionally supportive and effective learning environment (Toyama and Yamazaki, 2021). Besides, it is important to note that future related research based on control-value theory needs to integrate skill-specific assessment mechanisms (Pekrun et al., 2007), as writing anxiety has unique control appraisals (e.g., orthographic processing: Rasool et al., 2023a), distinguishing from speaking anxiety.

In addition, the moderating effects of language level and gender suggest the need for differentiated instructional support tailored to specific learner profiles. For beginners, instructional design should focus on reducing cognitive load through chunked character learning and scaffolding strategies (Sweller, 2020). Furthermore, for female learners, anxiety can be alleviated through activities that help reframe errors as opportunities for growth rather than failure (Rasool et al., 2023a, 2023b). For advanced learners, simulated pragmatic contexts can be integrated into instruction to address the complexity of sociolinguistic and discourse-level demands, thereby enhancing control appraisals and reducing anxiety (Dewaele et al., 2018).

Several limitations of this study should be acknowledged. First, the majority of primary studies included in this meta-analysis relied on self-report instruments to measure foreign language anxiety. While such tools are widely adopted in affective research due to their practicality, they are prone to response biases, including social desirability effects and inaccuracies in self-perception. Second, the exclusive use of single-source questionnaire data raises the possibility of common-method bias, such as item context effects, whereby responses to earlier items may influence subsequent answers. Third, few studies differentiated between anxiety subtypes (e.g., writing-specific vs. speaking-specific), which limits the analytical granularity and reduces the interpretability of skill-based variation. Fourth, the included pre-existing studies are objectively limited in their exploration of some groups. For example, African and Oceanian samples remain critically underrepresented, which may limit the understanding of these groups.

To address these limitations, future research should move forward in three key directions. First, multimodal assessment strategies—such as combining self-report measures with physiological indicators (e.g., heart rate variability), behavioral data (e.g., avoidance patterns), or observational methods—should be more widely adopted. Integrating these complementary approaches would enhance the psychometric rigor of anxiety assessment and provide a more accurate and objective basis for understanding learners’ emotional experiences. Second, future meta-analytic studies should examine skill-specific dimensions of anxiety to capture how affective responses vary across different language modalities and task types. Third, greater attention should be given to learners from African and Oceanian regions, who remain significantly underrepresented in the existing literature. Expanding research in these contexts is crucial for developing more equitable and generalizable insights into second language learning.

## Data Availability

The original contributions presented in the study are included in the article/Supplementary material, further inquiries can be directed to the corresponding author/s.

## References

[ref1] An H. Li S. (2024). Task-specific writing anxiety and self-efficacy are separate from general L2 writing anxiety and self-efficacy and they have differential associations with the effects of written corrective feedback in pre-task and within-task planning. System 126:103480. doi: 10.1016/j.system.2024.103480

[ref2] Baran-Łucarz M. (2022). “Language anxiety” in The Routledge handbook of second language acquisition and speaking (Milton Park, Abingdon, Oxfordshire, UK: Routledge), 83–96.

[ref3] Basith A. Musyafak N. Ichwanto M. A. Syahputra A. (2019). Chinese learning anxiety on foreign students. Eur. J. Educ. Res. 8, 1193–1200.

[ref4] Chang A. C. S. (2008). Sources of listening anxiety in learning English as a foreign language. Percept. Mot. Skills 106, 21–34. doi: 10.2466/pms.106.1.21-34, PMID: 18459352

[ref5] Chen J. Y. (2018). A review of research on Chinese language learning anxiety: a statistical analysis of journal articles from 1999 to 2017. Educ Modernization. 5, 313–316.

[ref6] Chen W. (2025). Systematic review and meta-analysis of the relationship between foreign language anxiety and academic achievement in Chinese language learners. Front. Educ. 10:1576224. doi: 10.3389/feduc.2025.1576224

[ref7] Demirdöken G. Okur S. (2022). Psychometric properties of speaking anxiety scale and an interdisciplinary investigation with serial mediation analysis. Innov. Lang. Learn. Teach. 17, 706–722. doi: 10.1080/17501229.2022.2123920

[ref8] Dewaele J. M. MacIntyre P. D. Boudreau C. Dewaele L. (2016). Do girls have all the fun? Anxiety and enjoyment in the foreign language classroom. Theory Pract. Second Lang. Acquis. 2, 41–63.

[ref9] Dewaele J. M. Witney J. Saito K. Dewaele L. (2018). Foreign language enjoyment and anxiety: the effect of teacher and learner variables. Lang. Teach. Res. 22, 676–697. doi: 10.1177/1362168817692161

[ref10] Egger M. Zellweger-Zähner T. Schneider M. Junker C. Lengeler C. Antes G. (1997). Language bias in randomised controlled trials published in English and German. Lancet 350, 326–329. doi: 10.1016/S0140-6736(97)02419-7, PMID: 9251637

[ref11] Fan Z. (2009). The anxiety sources of Chinese reading in various countries. Chin Lang Learn. 6, 99–105.

[ref12] Ghorbandordinejad F. (2014). Examining the relationship between students’ levels of perfectionism and their achievements in English learning. Res. Engl. Lang. Pedagogy 2, 36–45.

[ref13] Grégoire G. Derderian F. Le Lorier J. (1995). Selecting the language of the publications included in a meta-analysis: is there a tower of babel bias? J. Clin. Epidemiol. 48, 159–163. doi: 10.1016/0895-4356(94)00098-B, PMID: 7853041

[ref14] Horwitz E. K. (2016). Factor structure of the foreign language classroom anxiety scale: comment on Park (2014). Psychol. Rep. 119, 71–76. doi: 10.1177/0033294116653368, PMID: 27287268

[ref15] Li C. Dewaele J. M. (2021). How classroom environment and general grit predict foreign language classroom anxiety of Chinese EFL students. J. Psychol. Lang. Learn. 3, 86–98. doi: 10.52598/jpll/3/2/6

[ref16] Li S. Wang H. (2018). “Traditional literature review and research synthesis” in The Palgrave handbook of applied linguistics research methodology, 123–144.

[ref17] Liang Z. H. Quan K. L. (2016). Classroom learning anxiety and its alleviation among ASEAN international students in China. J Guangxi Norm Univ (Philos Soc Sci Ed). 52, 126–131.

[ref18] Lipsey M. W. Wilson D. B. (2001). Practical meta-analysis. Thousand Oaks, CA: SAGE Publications.

[ref19] Little R. J. Rubin D. B. (2019). Statistical analysis with missing data. 3rd Edn. Hoboken, NJ: John Wiley & Sons.

[ref20] Liu M. (2018). Bilingual/multilingual learners’ willingness to communicate in and anxiety on speaking Chinese and their associations with self-rated proficiency in Chinese. Int. J. Bilingual Educ. Bilingualism 21, 54–69. doi: 10.1080/13670050.2015.1127889

[ref21] Luo H. (2015). Chinese language learning anxiety: a study of heritage learners. Herit. Lang. J. 12, 22–47. doi: 10.46538/hlj.12.1.2

[ref22] Norris J. M. Ortega L. (2006). “The value and practice of research synthesis for language learning and teaching” in Synthesizing research on language learning and teaching (Amsterdam: John Benjamins), 350.

[ref23] Oswald F. L. Plonsky L. (2010). Meta-analysis in second language research: choices and challenges. Annu. Rev. Appl. Linguist. 30, 85–110. doi: 10.1017/S0267190510000115

[ref24] Park G. P. French B. F. (2013). Gender differences in the foreign language classroom anxiety scale. System 41, 462–471. doi: 10.1016/j.system.2013.04.001

[ref25] Patsopoulos N. A. Evangelou E. Ioannidis J. P. (2008). Sensitivity of between-study heterogeneity in meta-analysis: proposed metrics and empirical evaluation. Int. J. Epidemiol. 37, 1148–1157. doi: 10.1093/ije/dyn065, PMID: 18424475 PMC6281381

[ref26] Pekrun R. Frenzel A. C. Goetz T. Perry R. P. (2007). “The control-value theory of achievement emotions: An integrative approach to emotions in education” in Emotion in education. eds. Schutz P. A. Pekrun R. (San Diego, CA: Academic Press), 13–36.

[ref27] People’s Daily Overseas Edition (2022). 76 countries have incorporated Chinese into their national education systems [internet]. Beijing: Ministry of Commerce of the People’s Republic of China.

[ref28] Perera H. N. DiGiacomo M. (2013). The relationship of trait emotional intelligence with academic performance: a meta-analytic review. Learn. Individ. Differ. 28, 20–33. doi: 10.1016/j.lindif.2013.08.002

[ref29] Piniel K. Zólyomi A. (2022). Gender differences in foreign language classroom anxiety: results of a meta-analysis. Stud. Second Lang. Learn. Teach. 12, 173–203. doi: 10.14746/ssllt.2022.12.2.2

[ref30] Plonsky L. Brown D. (2015). Domain definition and search techniques in meta-analyses of L2 research (or why 18 meta-analyses of feedback have different results). Second. Lang. Res. 31, 267–278. doi: 10.1177/0267658314536436

[ref31] Plonsky L. Oswald F. L. (2014). How big is “big”? Interpreting effect sizes in L2 research. Lang. Learn. 64, 878–912. doi: 10.1111/lang.12079

[ref32] Plonsky L. Oswald F. L. (2015). “Meta-analyzing second language research” in Advancing quantitative methods in second language research. ed. Plonsky L. (New York: Routledge), 106–128.

[ref33] Rasool U. Aslam M. Z. Mahmood R. Barzani S. H. H. Qian J. (2023a). Pre-service EFL teacher’s perceptions of foreign language writing anxiety and some associated factors. Heliyon 9:e13405. doi: 10.1016/j.heliyon.2023.e13405, PMID: 36816292 PMC9932654

[ref34] Rasool U. Qian J. Aslam M. Z. (2023b). An investigation of foreign language writing anxiety and its reasons among pre-service EFL teachers in Pakistan. Front. Psychol. 13:8157. doi: 10.3389/fpsyg.2022.947867, PMID: 36687864 PMC9848586

[ref35] Rothstein H. R. (2008). Publication bias as a threat to the validity of meta-analytic results. J. Exp. Criminol. 4, 61–81. doi: 10.1007/s11292-007-9046-9

[ref36] Spielberger C. D. (1983). State-trait anxiety inventory for adults (STAI-AD) [database record]. APA PsycTests.

[ref37] Stankov L. (2010). Unforgiving Confucian culture: a breeding ground for high academic achievement, test anxiety and self-doubt? Learn. Individ. Differ. 20, 555–563. doi: 10.1016/j.lindif.2010.05.003

[ref38] Sweller J. (2020). Cognitive load theory and educational technology. Educ. Technol. Res. Dev. 68, 1–16. doi: 10.1007/s11423-019-09701-3

[ref39] Teimouri Y. Goetze J. Plonsky L. (2019). Second language anxiety and achievement: a meta-analysis. Stud. Second. Lang. Acquis. 41, 363–387. doi: 10.1017/S0272263118000311

[ref40] Terrin N. Schmid C. H. Lau J. Olkin I. (2003). Adjusting for publication bias in the presence of heterogeneity. Stat. Med. 22, 2113–2126. doi: 10.1002/sim.1461, PMID: 12820277

[ref41] Toyama M. Yamazaki Y. (2021). Classroom interventions and foreign language anxiety: a systematic review with narrative approach. Front. Psychol. 12:614184. doi: 10.3389/fpsyg.2021.614184, PMID: 33633643 PMC7900487

[ref42] Vuogan A. Li S. (2023). A systematic review of meta-analyses in second language research: current practices, issues, and recommendations. Appl. Linguist. Rev. 15, 1621–1644. doi: 10.1515/applirev-2023-0000

[ref43] Wang Q. Du F. (2020). Anxiety in Chinese language learning: relationship to motivation, attitude, and achievement. Electron. J. Res. Educ. Psychol. 18, 447–472. doi: 10.25115/ejrep.v18i52.3180

[ref44] Wang Q. Li S. East M. (2024). Measuring L1 Chinese speakers’ anxiety when completing an English as L2 video narration task: validation of task anxiety and pedagogical implications. J. Second Lang. Stud. 7, 99–128. doi: 10.1075/jsls.00027.wan

[ref45] Xu (2018). A review of research on Chinese language learning anxiety in the past decade. J Chongqing Second Norm Univ. 31, 113–117.

[ref46] Yang L J. (2010). A study on Chinese language learning anxiety among Thai international students [dissertation]. Chongqing: Southwest University

[ref47] Yao S. Zhang D. Shen Q. (2022). Research on anxiety of learning Chinese as a second or foreign language in and outside mainland China: a systematic review of the literature 1999–2020. Front. Psychol. 13:843858. doi: 10.3389/fpsyg.2022.843858, PMID: 35360624 PMC8960163

[ref48] Zhang L. Wang B. (2002). Analysis of the correlation between international students’ Chinese language anxiety and academic performance, and teaching strategies. Lang. Teach. Res. 1, 36–42.

[ref49] Zhang R. F. Yang Y. S. (2011). Correlation analysis between international students’ Chinese learning anxiety and HSK test scores. J. Inner Mongolia Norm. Univ. (Nat Sci Ed) 40, 212–216.

[ref50] Zhou J. Y. (2023). The correlation between affective factors and oral Chinese proficiency among international students [master’s thesis]. Zhengzhou: Zhengzhou University.

