# Supplementary figures and images for: The impact of anxiety on Chinese as a second language achievement: a meta-analytic perspective
Source: Front Psychol. 2025 Sep 15;16:1620275. doi: 10.3389/fpsyg.2025.1620275 (PMC12477158; doi:10.3389/fpsyg.2025.1620275)

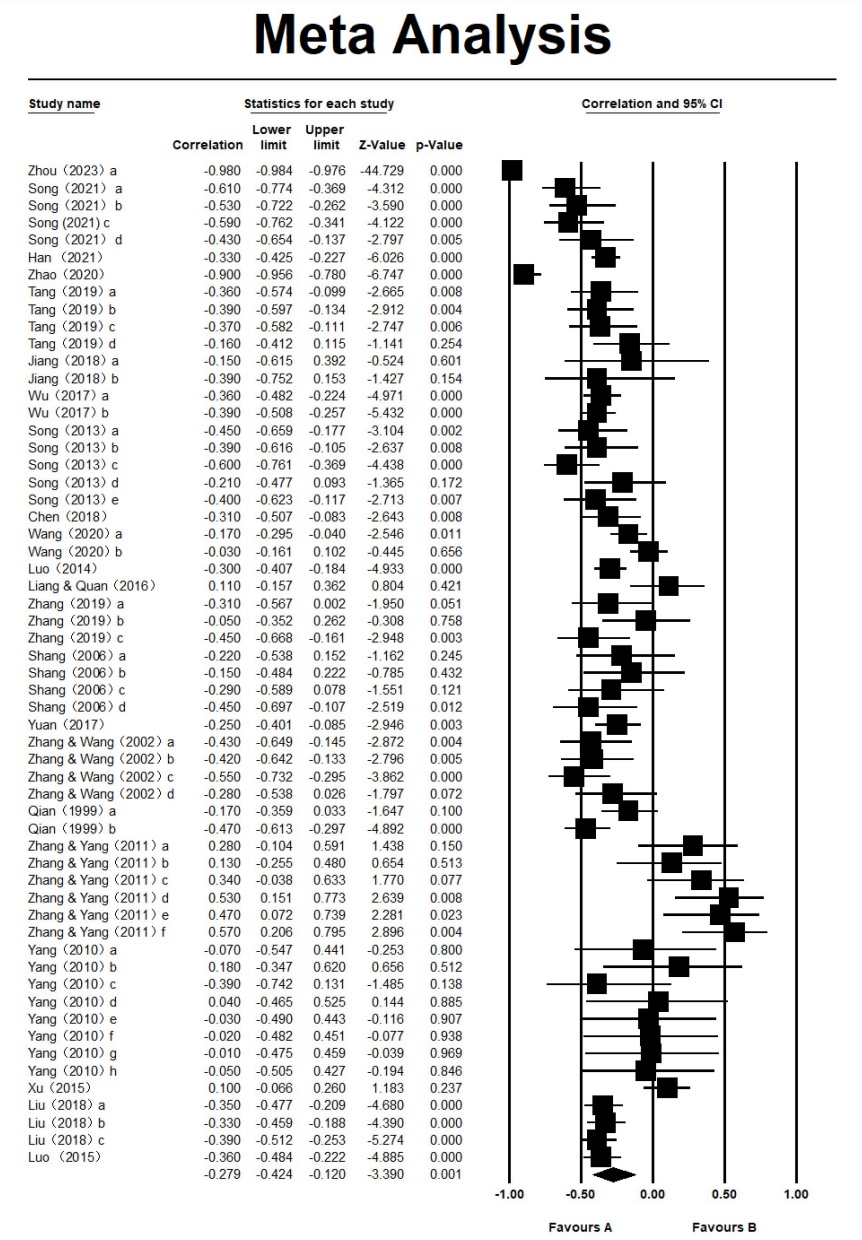

Supplement: Supplementary file 1 [file Image_1.jpeg]

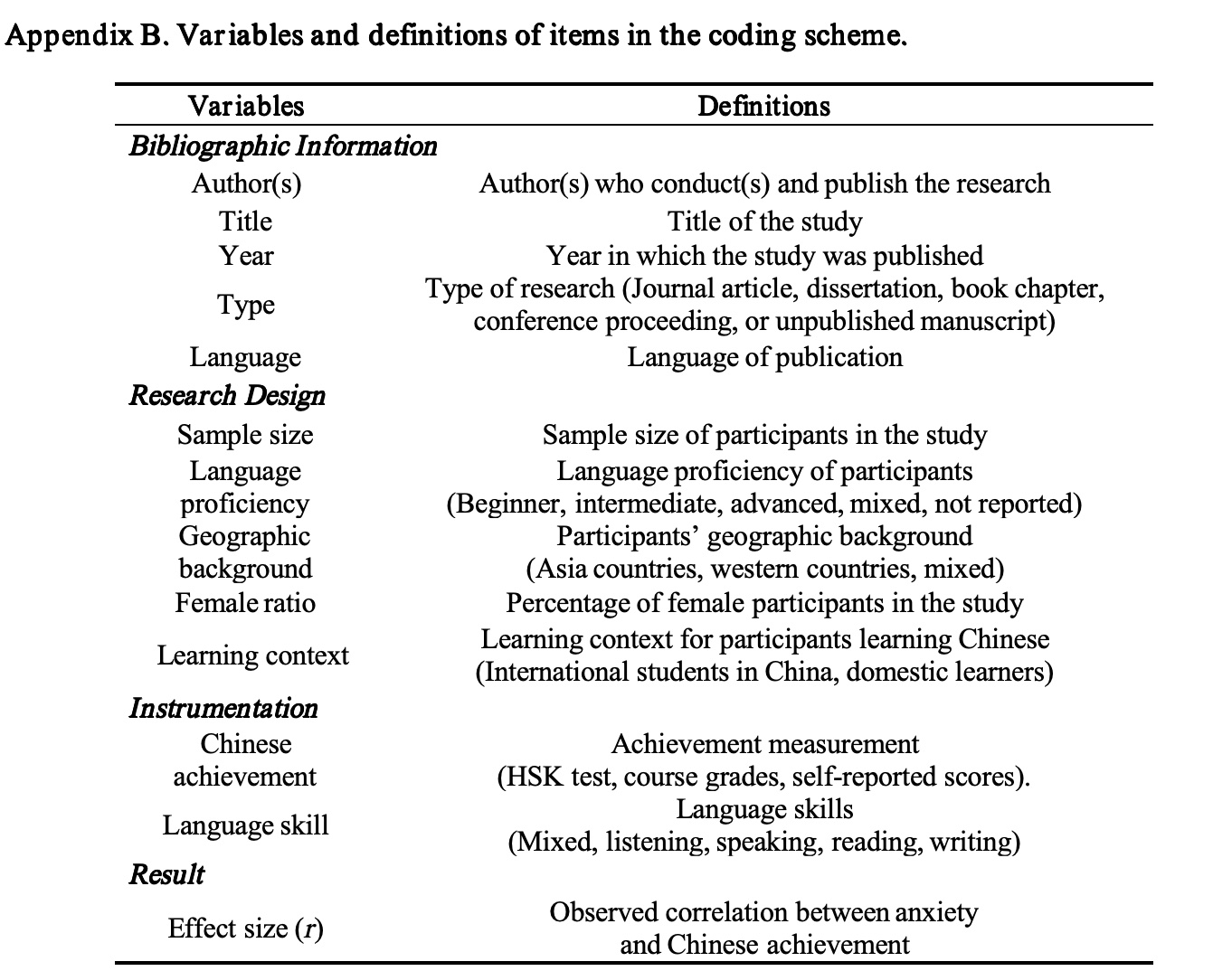

Supplement: Supplementary file 2 [file Image_2.jpeg]
